# GGPS1 Promoter Variant (rs3806394) Is Associated With Larger Simple Renal Cysts via Reduced GGPPS Expression

**DOI:** 10.1155/humu/8347509

**Published:** 2026-03-05

**Authors:** Kai Wang, Tianzhen Tang, Tao Xu, Zhen Chen, Liming Gou, Yuqi Wang, Dong Wang, Jing Wu, Xiuxing Wang, Bin Xue, Xianlin Xu

**Affiliations:** ^1^ Department of Urology, Sir Run Run Hospital, Nanjing Medical University, Nanjing, Jiangsu Province, China, njmu.edu.cn; ^2^ Institute for Brain Tumors, Jiangsu Provincial Key Laboratory of Cancer Biomarkers, Prevention and Treatment, Collaborative Innovation Center for Cancer Personalized Medicine, Nanjing Medical University, Nanjing, Jiangsu Province, China, njmu.edu.cn; ^3^ Department of Nephrology, State Key Laboratory of Reproductive Medicine, Children′s Hospital of Nanjing Medical University, Nanjing, Jiangsu Province, China; ^4^ Department of Core Laboratory, Sir Run Run Hospital, Nanjing Medical University, Nanjing, Jiangsu Province, China, njmu.edu.cn; ^5^ Department of Urology, The Third Affiliated Hospital of Soochow University, Changzhou, Jiangsu Province, China, suda.edu.cn

**Keywords:** geranylgeranyl pyrophosphate synthase, ggps1, rs3806394, simple renal cysts, SNPs

## Abstract

**Objective:**

We are aimed at investigating the association between the size of simple renal cyst (SRC) and the expression of geranylgeranyl pyrophosphate synthase (GGPPS), which can induce renal cyst formation after its deletion.

**Methods:**

Seventy‐seven patients who received renal cyst decortication were enrolled. Clinical characteristics and tissue sections were collected. We used immunohistochemistry and immunofluorescence to detect the expression and location of GGPPS in SRC tissues. The relationship between GGPPS expression and renal cyst size was evaluated by multivariate linear regression analysis. The tagging SNPs located in GGPS1 promoter were identified and the effect of the rs3806394 locus variant on GGPS1 promoter activity was assessed.

**Results:**

Immunohistochemistry and Western blot analysis revealed that GGPPS expression was downregulated in SRC tissues and that the larger the renal cyst volume was, the lower the expression of GGPPS in the cyst lining epithelial. Multivariate linear regression analysis indicated that low GGPPS levels in SRCs were independently related to large SRC size. Additionally, we reported that the frequency of the rs3806394 variant located in the GGPS1 promoter increased in SRC patients. The variant of the rs3806394 locus could decrease the activity of the GGPS1 promoter.

**Conclusion:**

A reduction in GGPPS expression in the cyst lining epithelium was associated with a risk of larger SRC size. The variant of the rs3806394 locus may be one of the reasons for the differential GGPPS expression in SRC tissues among the patients. These findings offer novel insights into the pathological mechanisms of SRC development.

## 1. Introduction

Simple renal cyst (SRC) is the most common type of acquired renal cysts in the adult population. The prevalence of this disease is considered to be approximately 7%–10% [1]. The incidence rate of SRC also increases with age [2]. Male sex, hypertension, and renal dysfunction have been reported to be associated with an increased incidence of SRC [3]. Less attention has been given to SRC in clinical practice unless symptoms such as abdominal discomfort, flank pain, hematuria, infection, or rupture are present because of complications or cyst size [4]. With these symptoms, intervention for SRC is considered necessary. In addition, researchers have increasingly reported that SRC is associated with hypertension, albuminuria, renal function, and early and long‐term allograft function [5–8].

Most SRCs probably remain stable in size and features over long periods of time, but sometimes the cyst may rapidly increase in size, especially in younger patients [9]. However, the pathological mechanism of SRC formation remains unclear. One hypothesis is that compensatory hyperfiltration and tubular hypertrophy due to parenchymal ischemia contribute to cyst formation [10]. Other research has speculated that SRC originates from a diverticulum on distal and collecting tubules [11].

In our previous study, we unexpectedly found that specific *Ggps1* gene deletion in renal tubular epithelial cells in a mouse model induced the formation and expansion of renal cysts [12]. The downregulation of geranylgeranyl pyrophosphate synthase (GGPPS), which is encoded by the *GGPS1* gene in renal tubular epithelial cells, also promotes HK2 cell proliferation. GGPPS is a key enzyme in the isoprenoid biosynthesis pathway, and its expression affects posttranslational isoprene modification of proteins [13]. Aberrant GGPPS expression has been linked to a variety of human diseases, such as liver disease, Type 2 diabetes, lung disease, and various malignant tumors [13]. In the present work, we are aimed at investigating the association between GGPPS expression and the development of SRC.

## 2. Materials and Methods

### 2.1. Study Population

We retrospectively collected data from the patients with SRC who received renal cyst decortication at Sir Run Run Hospital Nanjing Medical University (Nanjing, China) from January 2016 to June 2022, which was performed according to the criteria of the STROBE guidelines [14]. The diagnosis of SRC was based on pathology. Patients with concurrent renal carcinoma or polycystic kidney disease were excluded. We also excluded the patients whose data were incomplete. Normal kidney tissues were obtained from the nontumorous, histologically normal renal parenchyma of patients who underwent radical nephrectomy for renal cell carcinoma. These samples were taken from areas remote from the tumor and confirmed by pathology to be free of malignant or inflammatory changes.

This study was carried out in accordance with the Declaration of Helsinki, and the protocol was approved by the Ethics Committee of Sir Run Run Hospital Nanjing Medical University (No. 2021‐SR‐036). Written informed consent was obtained from every patient enrolled in the study.

### 2.2. Data Collection and Definitions

The data of clinical characteristics, relevant laboratory indicators and complications were retrospectively collected from medical records. The number and location of the cysts and the diameter of the largest cyst were confirmed by computed tomography scan before surgery.

Hypertension was defined as reported, antihypertensive treatment, a systolic blood pressure ≥ 140 mmHg or a diastolic blood pressure ≥ 90 mmHg. Diabetes mellitus was defined as reported, hypoglycemic therapy, or fasting glycemia ≥ 7 mmol/L. Chronic kidney disease (CKD) was defined as an estimated eGFR < 60 mL/min per 1.73m^2^ [15]. The volume of the largest cyst was calculated according to the formula: *V* = 4/3∗*π*∗(length/2)∗(width/2)∗(height/2).

### 2.3. Histopathology, Immunohistochemistry (IHC), and Immunofluorescence (IF)

The tissue sections were obtained retrospectively from residual tissue wax stored in the pathology department of Sir Run Run Hospital Nanjing Medical University. The sections were cut into 4‐*μ*m sections. For IHC and IF staining, the sections were deparaffinized, rehydrated, antigen‐retrieved, blocked, and subsequently incubated with primary and secondary antibodies according to a standard protocol. The following antibody was used: anti‐GGPPS (#sc‐271680, Santa Cruz Biotechnology, Santa Cruz, United States). The stain percentage results were analyzed by Image J software (National Institute of Heath, Bethesda, United States).

### 2.4. Western Blot

Western blot assays were performed as described in our previous study [12]. Briefly, total protein was extracted with RIPA lysis buffer. Equal amounts of protein were separated on 10% SDS–polyacrylamide gels and subsequently transferred onto PVDF membranes. After blocking with 5% skim milk in TBST, the membranes were incubated with the appropriate primary antibodies, followed by incubation with horseradish peroxidase–conjugated secondary antibodies. Immunoreactive bands were visualized using enhanced chemiluminescence (ECL) (Thermo, United States). The antibodies used in this study were: anti‐GGPPS (#sc‐271680, Santa Cruz Biotechnology) and anti‐*β*‐actin (#BS1002, Bioworld Technology, United States).

### 2.5. Selection of Single‐Nucleotide Polymorphisms (SNPs) in the GGPS1 Promoter

The data of Han Chinese in Beijing (CHB) and Southern Han Chinese (CHS) from the 1000 Genomes Project (https://www.internationalgenome.org/) were used to identify SNPs in the *GGPS1* promoter. Next, tagging SNPs matching the following quality control criteria were selected: minimum minor allele frequency (MAF) >0.05, minimum genotype minimum > 95*%*, and *p* value of Hardy–Weinberg equilibrium (HWE) > 0.001. We subsequently used Haploview 4.2 software to perform pairwise linkage disequilibrium (LD) analysis (*R* ^2^ ≥ 0.8) to select tagging SNPs. The reference allele and alt allele frequencies of SNPs in East Asians based on the 1000 Genomes Project were obtained from dbSNP Overview (https://www.ncbi.nlm.nih.gov/snp/).

### 2.6. SNP Genotyping Identification

Genomic DNA was extracted from the venous blood of all patients using the DNeasy Blood & Tissue Kits (TIANGEN, China). Probe‐based quantitative real‐time polymerase chain reaction (qRT‐PCR) (TaqMan 5 ^′^‐nuclease allelic discrimination) was performed to identify SNPs using a kit from Biocolors Corporation (Shanghai, China). The following primers were used in this study: rs6688441, F1 5 ^′^‐AGCCACAGCTTTTACCTTCTACAAA‐3 ^′^, F2 5 ^′^‐AGCCACAGCTTTTACCTTCTACAAC‐3 ^′^, and R 5 ^′^‐CAAATTAAAATTTGTAATACTTGTG‐3 ^′^; and rs3806394, F1 5 ^′^‐TGATGAAAGAGAATCTTATTTATTA‐3 ^′^, F2 5 ^′^‐TGATGAAAGAGAATCTTATTTATTG‐3 ^′^, and R 5 ^′^‐CTAAAATTTTACAGTACTGTTCACA‐3 ^′^. The sample size was determined on the basis of pre‐experiment data using PASS 2021 software (NCSS, United States). With a significance level of 0.05 and a power of 80%, the sample size was initially calculated and then increased by 10% to accommodate potential variability and dropouts, yielding a final sample size of 20 subjects per group.

### 2.7. Plasmid Construction and Luciferase Reporter Assay

The transcription factor FOXA1 was predicted to bind to the rs3806394 locus by JASPAR software (https://jaspar.genereg.net/). The recombinant plasmids pGL4.10–*GGPS1* promoter (WT), pGL4.10‐*GGPS1* promoter (MUT), and FOXA1–plasmid were constructed by OBiO Technology Company (Shanghai, China). pGL4.10–Empty was used as control. Differences in promoter activity caused by variation (*C* > *T*) in the rs3806394 alleles of the *GGPS1* gene were measured by a luciferase reporter assay. The human renal tubular epithelial cell line HK2 was maintained in RPMI‐1640 medium (Invitrogen, Shanghai, China) supplemented with 10% fetal bovine serum (Cyagen Biosciences, Shanghai, China) and 1% penicillin–streptomycin at 37°C in a humidified incubator with 5% CO_2_. For the luciferase reporter assay, HK2 cells were seeded in 12‐well plates and transfected with 0.1 *μ*g/mL pGL4.10–*GGPS1* promoter (WT) or promoter (MUT) plasmids using Lipofectamine 2000 (Invitrogen) at 60% confluence for 48 h. The cells were simultaneously transfected with 0.1 *μ*g/mL FOXA1 plasmid and 0.01 *μ*g/mL Renilla plasmid. Afterward, the transfected cells were collected, and firefly luciferase activity was detected by a Dual Luciferase Reporter Assay System (Promega, Beijing, China). Renilla luciferase activity was used as an internal reference.

### 2.8. Chromatin Immunoprecipitation Quantitative PCR (ChIP–qPCR)

To investigate the binding of FOXA1 to the predicted region containing rs3806394, we performed ChIP‐qPCR according to the instructions of the ChIP Assay Kit (Beyotime, China). Briefly, HK2 cells were cross‐linked with 1% formaldehyde and lysed in ChIP lysis buffer. Genomic DNA was sheared into fragments of ~400–800 bp by sonication and incubated overnight with anti‐FOXA1 antibody (#20411‐1‐AP, Proteintech, China) or IgG (#30000‐0‐AP, Proteintech) bound to magnetic beads. After being washed to remove nonspecific bindings, the protein–DNA complexes were eluted and reverse‐crosslinked. The DNA was purified and analyzed by qRT‐PCR using primers targeting the rs3806394 region. The data were statistically analyzed to assess differences in FOXA1 binding. The following primers were used: rs3806394, F 5 ^′^‐TGACGAGTCAAAGAGTTTTCTGC‐3 ^′^, and R 5 ^′^‐TCTGACCCTAGAAACTGAACTGT‐3 ^′^; and the GAPDH promoter, F 5 ^′^‐TACTAGCGGTTTTACGGGCG‐3 ^′^, and R 5 ^′^‐ TCGAACAGGAGGAGCAGAGAGCGA‐3 ^′^.

### 2.9. Statistical Analysis

Continuous variables with normal distribution were expressed as mean ± SD, whereas the variables with skewed distribution were expressed as the median (IQR). The categorical variables are expressed as numbers and frequencies. Eventually, *t*‐test or nonparametric test was subsequently conducted to compare continuous variables with normal or skewed distributions, respectively. Categorical variables were compared by the chi‐square test. Linear regression analysis and binary logistic regression methodology were used for univariate and multivariate analyses. All the statistical tests were performed using SPSS Statistics 23.0 software (IBM, New York, United States). Two‐sided *p* values < 0.05 were considered to indicate statistical significance.

## 3. Results

### 3.1. GGPPS Expression Is Downregulated in SRC Tissues

In our previous study, we reported that specific *Ggps1* gene deletion in renal tubular epithelial cells in a mouse model induced the formation of renal cysts [12]. The knockdown of GGPPS in HK2 cells increased their proliferation ability [12]. We first detected the expression of GGPPS in SRC tissues and normal kidney tissues by IF and IHC. GGPPS was specifically expressed in human renal tubular epithelial cells (Figure 1a). These results are consistent with our previous findings. We also found that GGPPS staining was significantly weaker in SRC tissues than in normal kidney tissues (Figure 1b). In addition, Western blot analysis confirmed this difference in GGPPS expression between normal kidney and SRC tissues (Figure 1c). These results indicated that GGPPS may contribute to the SRC development.

Figure 1GGPPS expression decreased in human SRC tissues. (a) GGPPS expression was detected in normal kidney and SRC tissues by IF, followed by confocal microscopy. GGPPS expression is shown in red, and nuclear staining was performed with DAPI (blue). (b) IHC was used to detect GGPPS expression in normal kidney and SRC tissues (*n* = 5). The GGPPS positive area (%) was determined using ImageJ software. (c) Western blot analysis of GGPPS expression in normal kidney and SRC tissues (*n* = 5). The protein expression of GGPPS was normalized to that of *β*‐actin.

**Figure:**
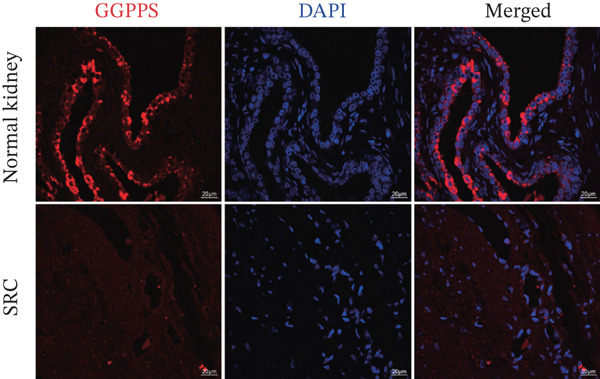
(a)

**Figure:**
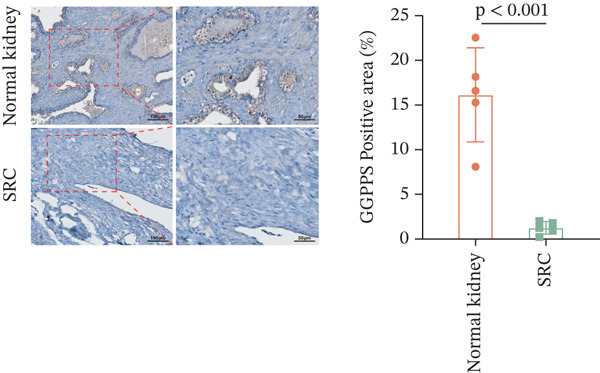
(b)

**Figure:**
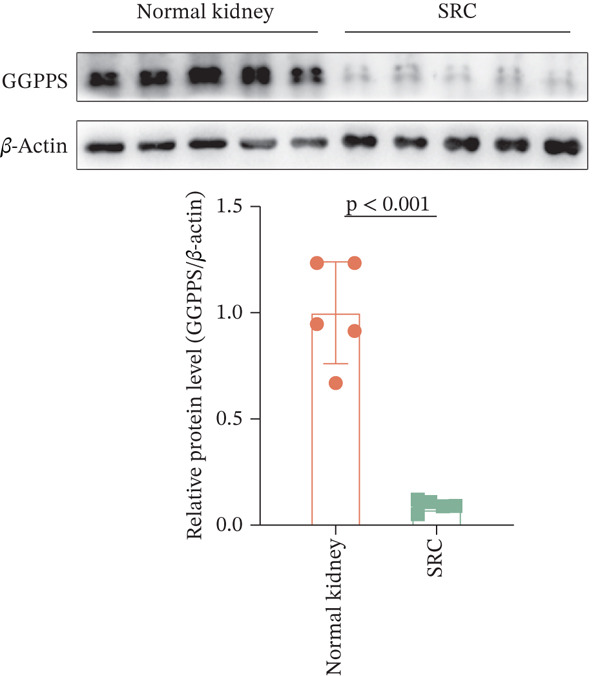
(c)

### 3.2. Low GGPPS Expression Is Associated With Larger Size of the SRC

To further determine the role of GGPPS in the development and size of SRC, we retrospectively analyzed data from 77 patients with SRC. We divided these patients into groups with larger cysts (*n* = 38) and smaller cysts (*n* = 39) with a median cutoff based on the volume of the largest cyst. The clinical characteristics of the patients are summarized in Table 1. Among these 77 patients, 40 (51.9%) were males and 37 (48.1%) were ≥ 60 years old. The median and average age was 59 (21–78) and 57.6 years old, respectively. The median volume of the largest cyst was 86.7 cm^3^. As shown in Table 1, the patients with a relatively large volume of the largest cyst were relatively older (*p* = 0.049). GGPPS expression in SRC tissues was also lower in patients with larger cysts (*p* = 0.007). In addition, there were no differences between the two groups in terms of gender, BMI, blood pressure, number and location of cysts, hypertension, diabetes, CKD, or nephrolith.

**Table 1:** Characteristics of the patients according to volume of simple renal cysts.

Variables	Cyst volume		*p* value
	Smaller (*n* = 39)	Larger (*n* = 38)	
Age (years)	55.2 ± 13.0	60.1 ± 8.2	0.049∗
Gender			
Male (*n* = 40)	17 (43.6%)	23 (60.5%)	0.137
Female (*n* = 37)	22 (56.4%)	15 (39.5%)	
BMI (kg/m^2^)	25.0 ± 3.3	24.2 ± 3.1	0.331
Systolic BP (mmHg)	133.7 ± 19.4	133.0 ± 15.8	0.864
Diastolic BP (mmHg)	83.7 ± 11.3	84.2 ± 9.6	0.812
Number of cysts			
1 (*n* = 36)	19 (48.7%)	17 (44.7%)	0.726
≥ 2 (*n* = 41)	20 (51.3%)	21 (55.3%)	
Location			
Unilateral (*n* = 44)	23 (59.0%)	21 (55.3%)	0.742
Bilateral (*n* = 33)	16 (41.0%)	17 (44.7%)	
GGPPS expression (%)	0.554 (0.280–1.819)	0.260 (0.171–0.531)	0.007∗∗
Hypertension			
No (*n* = 44)	23 (59.0%)	21 (55.3%)	0.742
Yes (*n* = 33)	16 (41.0%)	17 (44.7%)	
Diabetes			
No (*n* = 65)	33 (84.6%)	32 (84.2%)	0.961
Yes (*n* = 12)	6 (15.4%)	6 (15.8%)	
CKD			
No (*n* = 73)	36 (92.3%)	37 (97.4%)	0.615
Yes (*n* = 4)	3 (7.7%)	1 (2.6%)	
Nephrolith			
No (*n* = 67)	34 (87.2%)	33 (86.8%)	0.965
Yes (*n* = 10)	5 (12.8%)	5 (13.2%)	

Abbreviations: BMI, body mass index; BP, blood pressure; CKD, chronic kidney disease.

^∗^ *p* < 0.05.

^∗∗^ *p* < 0.01.

### 3.3. Low GGPPS Expression Increased the Risk of the Large‐Sized SRC

**Figure 2:**
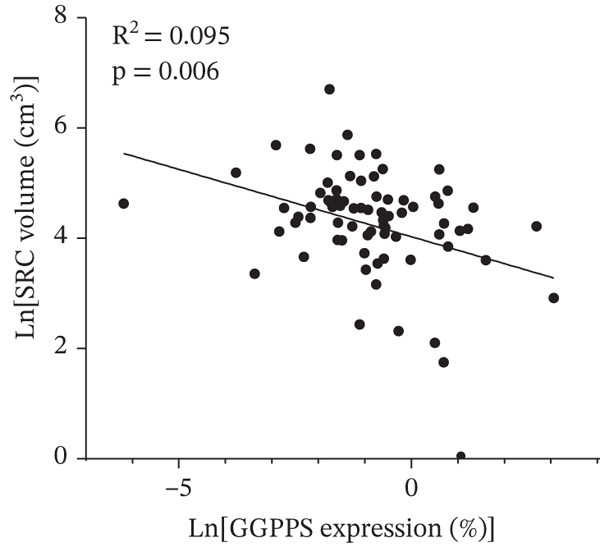
Scatter plot of the relationship between in‐transformed SRC volume (cm^3^) and in‐transformed GGPPS expression (%).

We subsequently investigated the association between GGPPS expression in SRC tissues and the volume of the cyst (Table 2). We converted continuous variables of GGPPS expression and cyst volume by Ln‐transformation in linear regression analysis. According to the univariate analysis, the *β*‐coefficient between the GGPPS expression level and cyst volume was −0.25 (95% CI: −0.42, −0.07; *p* = 0.006) (Figure 2). In the models adjusted for age, gender, and BMI, GGPPS expression remained negatively associated with cyst volume (*β* = −0.24; 95% CI: −0.42, −0.06; *p* = 0.009). In the multivariable models, GGPPS expression was still negatively associated with the size of cysts (*β* = −0.23; 95% CI −0.41, −0.04; *p* = 0.021), even when adjusted for age, gender, BMI, number and location of cysts, hypertension, diabetes, CKD, and kidney stones. Afterward, we performed a sensitivity analysis to verify the robustness of the results above. With respect to the continuous variable of GGPPS expression in SRC tissues, the patients were divided into low expression (*n* = 38) and high expression (*n* = 39) groups on the basis of the median value (Figure S1). Lower GGPPS expression was significantly related to larger cyst size (OR = 4.77, 95% CI 1.49–15.21; *p* = 0.008) when fully adjusting for covariates (Table S1). These results indicated that low GGPPS expression in SRC tissues is an independent risk factor for large cysts.

**Table 2:** The *β*‐coefficients for the association between GGPPS expression and simple renal cyst size.

Variable	Crude model		Model 1		Model 2	
	*β*‐Coefficients (95% CI)	*p* value	*β* ‐Coefficients (95% CI)	*p* value	*β*‐Coefficients (95% CI)	*p* value
Age (years)	−0.01 (−0.03, 0.02)	0.530	−0.01(−0.03, 0.02)	0.483	−0.01 (−0.04, 0.02)	0.533
Gender (female vs. male)	−0.28 (−0.79, 0.23)	0.280	−0.23(−0.73, 0.27)	0.357	−0.19 (−0.72, 0.34)	0.483
BMI (kg/m^2^)	0.01 (−0.07, −0.09)	0.768	−0.01(−0.10, 0.07)	0.751	−0.02 (−0.11, 0.07)	0.675
Systolic BP (mmHg)	0.00 (−0.01, 0.02)	0.865	—	—	—	—
Diastolic BP (mmHg)	0.00 (−0.02, 0.03)	0.897	—	—	—	—
Number of cysts (1 vs ≥2)	0.15 (−0.36, 0.67)	0.559	—	—	−0.00 (−0.82, −0.82)	0.996
Location (bilateral vs. unilateral)	0.15 (−0.37, 0.67)	0.587	—	—	0.23 (−0.52, 0.99)	0.538
Hypertension (yes vs. no)	0.32 (−0.20, 0.83)	0.224	—	—	0.18 (−0.42, 0.79)	0.548
Diabetes (yes vs. no)	−0.21 (−0.92, 0.49)	0.550	—	—	−0.34 (−1.09, 0.42)	0.374
CKD (yes vs. no)	−0.70 (−1.85, 0.45)	0.228	—	—	−0.67 (−1.87, 0.53)	0.271
Kidney stones (yes vs. no)	−0.04 (−0.81, 0.72)	0.915	—	—	−0.13 (−0.91, 0.65)	0.735
GGPPS expression (%)	−0.25 (−0.42, −0.07)	0.006	−0.24 (−0.42, −0.06)	0.009	−0.23 (−0.41, −0.04)	0.021

*Note:* Crude model was unadjusted for any covariates; Model 1 was adjusted for covariates including age, gender, and BMI; Model 2 was adjusted for covariates including age, gender, BMI, number of cysts, location of cysts, hypertension, diabetes, CKD, and kidney stones.

Abbreviations: BMI, body mass index; BP, blood pressure; CI, confidence interval; CKD, chronic kidney disease; OR, odds ratio.

### 3.4. Variant Frequency of rs3806394 Located in the GGPS1 Promoter Increased in SRC Patients

To explore the potential possibility of GGPPS expression downregulation, we collected venous blood samples from patients with SRC (*n* = 20) or without SRC (*n* = 20), and identified the SNPs located in the *GGPS1* promoter. We identified 11 variants in the promoter of *GGPS1* from the 1000 Genomes Project after quality control. After the LD analysis was performed, rs6688441 and rs3806394 were selected for further study (Figure 3a). Our results revealed that the numbers of SRC patients with the rs6688441 phenotype were 11 (55%), 6 (30%), and 3 (15%) for CC, CA, and AA, respectively (Table 3). The frequencies of the C and A alleles were 0.700 and 0.300, respectively. Moreover, the numbers of the rs6688441 phenotype in the patients without SRC were 13 (65%), 6 (30%), and 1 (5%) for CC, CA, and AA, respectively. The frequency of the A allele was 0.200, and the frequency of the C allele was 0.800 in participants without SRC, which did not differ from those with SRC (*p* = 0.772). The numbers of SRC patients with the rs3806394 phenotype were 1 (5%), 13 (65%), and 6 (30%) for CC, CT, and TT, respectively. The frequencies of the C and T alleles were 0.375 and 0.625, respectively. However, the numbers of the rs3806394 phenotype in the patients without SRC were 8 (40%), 11 (55%), and 1 (5%) for CC, CT, and TT, respectively. The T allele frequency was 0.325 in participants without SRC, which was significantly lower than those with SRC (*p* = 0.007). We also searched for the rs6688441 and rs3806394 phenotype among 1008 East Asian participants reported in the 1000 Genomes study. The A allele frequency of the rs6688441 phenotype was 0.251, and the T allele frequency of the rs3806394 phenotype was 0.228 in 1008 East Asian participants reported in 1000 Genomes study, respectively, which were similar to those observed in patients without SRC. These findings indicated that the variant frequency of rs6688441 in SRC patients is similar to that in participants without SRC and East Asian individuals, whereas the variant frequency of rs3806394 in SRC patients is significantly greater than that in participants without SRC and East Asian individuals. The variant of rs3806394 may contribute to the downregulation of GGPPS expression in SRC patients.

Figure 3The *GGPS1* rs3806394 variant is associated with gene transcription regulation. (a) Graphical representation of the localization of tagger SNP in the *GGPS1* promoter region. (b) The transcription factor FOXA1 was predicted to bind to the rs3806394 locus by JASPAR software (https://jaspar.genereg.net/). (c) ChIP was performed using an antibody against FOXA1 or normal IgG as a negative control. The recovered DNA was analyzed by qPCR using primers specific for the promoter region of GGPS1 which contained rs3806394 locus. (d) The relative luciferase activity was significantly reduced in HK2 cells transfected with the *GGPS1* promoter containing the T allele of rs3806394 (MUT) and the FOXA1 transcription factor plasmid after 48 h (*n* = 3).

**Figure:**
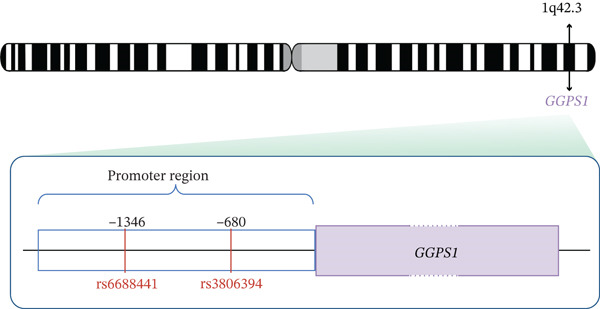
(a)

**Figure:**
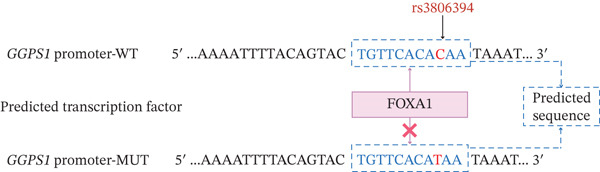
(b)

**Figure:**
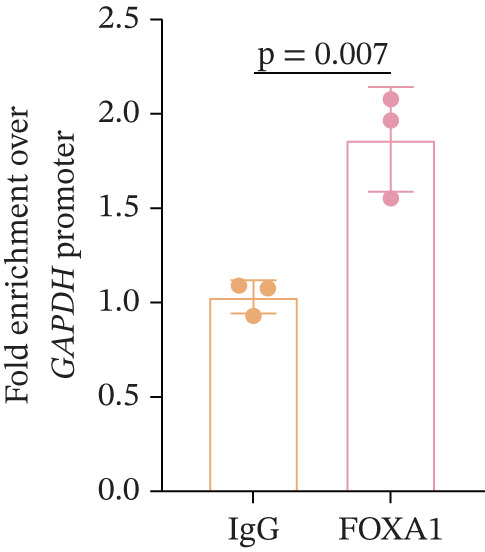
(c)

**Figure:**
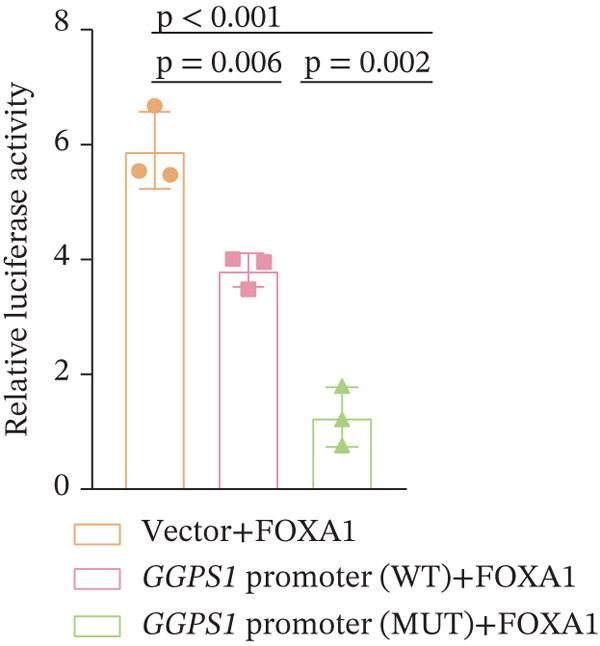
(d)

**Table 3:** SNPs genotype‐phenotype of *GGPS1* gene promoter in simple renal cyst patients.

Variant	Ch38p14	Location	Alleles	SRC	Frequency of SNPs genotype‐phenotype					*p* value
rs6688441	235325869	−1346	C: A	Yes (*n = 20*)	CC:0.550	CA:0.300	AA:0.150	C:0.700	A:0.300	0.772
				No (*n = 20*)	CC:0.650	CA:0.300	AA:0.050	C:0.800	A:0.200	
rs3806394	235326535	−680	C: T	Yes (*n = 20*)	CC:0.050	CT:0.650	TT:0.300	C:0.375	T:0.625	0.007
				No (*n = 20*)	CC:0.400	CT:0.550	TT:0.050	C:0.675	T:0.325	

Abbreviations: SRC, simple renal cyst; SNPs, single nucleotide polymorphisms.

To demonstrate the effect of the rs3806394 SNP on *GGPS1* promoter activity in renal tubular epithelial cells, we used *GGPS1* promoter reporters carrying either the C (WT) or the T (MUT) allele of this SNP and assessed reporter expression. The transcription factor FOXA1 was predicted to bind to the rs3806394 locus and this binding was affected by the T allele of this SNP (Figure 3b). The results of the ChIP‐qPCR assay validated this (Figure 3c). We observed that compared with cells transfected with the *GGPS1* promoter (WT) plasmid, HK2 cells transfected with the *GGPS1* promoter (MUT) plasmid had lower luciferase activity (Figure 3d). These results indicated that the T allele of rs3806394 in SRC patients is related to decreased *GGPS1* promoter activity.

## 4. Discussion

Our present research demonstrated that GGPPS was downregulated in SRC tissues and low GGPPS levels in SRC were an independent risk factor for the size of the SRC. A reduction in GGPPS in the cyst lining epithelial was associated with a larger SRC size. In addition, we report for the first time that the variant frequency of rs3806394, located in the promoter of *GGPS1*, is increased in SRC patients. The variant of the rs3806394 locus decreased the activity of the *GGPS1* promoter. This may explain why GGPPS expression is downregulated in most SRC patients.

The exact pathological mechanism underlying the occurrence and development of SRC is not well‐known. Most patients with SRC are incidentally diagnosed during imaging examination for other reasons [16]. The diverticulum on the distal nephron tubule is generally considered to be the likely starting point for cyst formation. Baert and Steg reported the formation of diverticula and microcysts in the kidney before the identification of SRC by imaging [11]. A nested case‐control study from China indicated that hyperuricemia could be independently associated with a high incidence of SRC in a nondiabetic population [17]. Serum uric acid levels are also positively correlated with the maximum cyst diameter. In addition, high parathormone levels, advanced age, and kidney stones are also independent risk factors for SRC in primary hyperparathyroidism patients [18]. With respect to the development of SRC, human SRC fluid samples can stimulate cyst formation in MDCK cells in vitro [19]. The results from our previous study also indicated that metabolic syndrome is associated with the SRC size [20]. Although some scholars believe that SRC could be a kind of variant “without known pathologic associations”, studies still indicate that SRC are related to the incidence of hypertension, kidney damage, and arteriosclerosis [21–24]. Thus, the management and treatment of SRC are still necessary for SRC patients.

Currently, there is no evidence linking the occurrence of SRC to any specific gene. However, several well‐established genetic determinants have been implicated in renal cystic disease [25]. Most cases of autosomal dominant polycystic kidney disease are attributed primarily to mutations in *PKD1* and *PKD2*, which encode polycystin‐1 and polycystin‐2, respectively. These proteins form a functional complex within the primary cilium to mediate mechanosensation and calcium signaling, and dysfunction of these proteins drives aberrant tubular proliferation and cyst expansion [26]. HNF1B is a transcription factor that regulates a broad spectrum of kidney development genes, such as *PKHD1*, *PKD2*, and *UMOD* [27]. Pathogenic variants in HNF1B are associated with renal cysts, congenital anomalies of the kidney and urinary tract, maturity‐onset diabetes of the young, and extrarenal phenotypes [28]. In contrast to these canonical cystogenic genes, GGPSS represents a novel metabolic regulator that acts through the disruption of isoprenoid biosynthesis and prenylation‐dependent signaling pathways. This highlights the genetic heterogeneity of renal cystogenesis, with contributions from both classical ciliary/tubular signaling pathways and newly recognized metabolic regulatory mechanisms.

As a key enzyme in the isoprenoid biosynthesis pathway, GGPPS is responsible for the synthesis of geranylgeranyl pyrophosphate from farnesyl pyrophosphate. Farnesyl pyrophosphate is the substrate for the farnesylation of proteins and the source for cholesterol synthesis, whereas geranylgeranyl pyrophosphate is the substrate for the geranylgeranylation of proteins as well as the precursor of vitamin K2 and ubiquinone [13]. Disruption of the balance between farnesylation and geranylgeranylation can influence the membrane localization and function of small GTPases, which play vital roles in various cell processes, such as cell proliferation, apoptosis, metabolism, differentiation, and protein trafficking [29]. In our previous work, we reported that GGPPS knockdown in HK2 cells increased cell proliferation ability [12]. This finding is consistent with our current results. Therefore, we believe that patients with lower GGPPS in renal cyst tissue tend to have larger cysts. Additionally, a variant of the rs3806394 locus may be one of the reasons for the differential GGPPS expression in SRC tissues among patients.

There are several limitations in our study. First, this research did not examine the detailed mechanisms responsible for SRC formation and development, and further molecular studies are needed. Second, the sample size used for SNP genotyping identification was relatively small and the effect of the SNP locus rs3806394 on GGPPS expression still needs to be further determined. Third, this study was performed among participants of a single race, and more research is needed to validate the results in a multiracial cohort. Fourth, this study was performed at a single time point, and causality could not be inferred. Fifth, although the probe‐based qPCR method remains the preferred approach for SNP genotyping, we recommend targeted resequencing in future studies to fine‐map additional variants in high LD. Finally, the SRC tissues used in this study were obtained from surgery, and the identification of GGPPS expression in smaller cyst tissues is lacking.

## 5. Conclusion

We described a novel expression pattern in which GGPPS is expressed in SRC tissues. We also showed that low GGPPS levels in the SRC were an independent risk factor for the large size of the SRC. Moreover, we report for the first time that the frequency of the rs3806394 variant located in the promoter of *GGPS1* is increased in SRC patients. Variants of this SNP locus are associated with decreased *GGPS1* promoter activity. These findings provide new insights into the pathological mechanism of SRC development.

## Author Contributions

K.W.: data curation, formal analysis, and writing—original draft; T.T.: data curation, formal analysis, and writing—original draft; T.X.: supervision; Z.C.: supervision; L.G.: resources; Y.W.: resources; D.W.: visualization; J.W.: project administration; X.W.: project administration; B.X.: conceptualization and writing—review and editing; X.X.: funding acquisition, project administration, conceptualization, and writing—review and editing. K.W., T.T., T.X., X.X., and Z.C. contributed equally to this work.

## Funding

This study was supported by the Clinical Research Special Fund of Wu Jieping Medical Foundation (320.6750.2024‐15‐29), Medical Research Funding Project of China Health and Medical Development Foundation, Beijing Bethune Charitable Foundation (2024‐YJ‐156‐J‐015), and National Natural Science Foundation of China (10.13039/501100001809) (32071142, 32271187).

## Disclosure

All authors reviewed the results and approved the final version of the manuscript.

## Conflicts of Interest

The authors declare no conflicts of interest.

## Supporting Information

Additional supporting information can be found online in the Supporting Information section.

## Supporting information


**Supporting Information 1** **Figure S1:** (a) Representative IHC staining images of GGPPS in SRC tissues. (b) Violin plot showing the SRC volume (cm^3^) for the low (*n* = 38) and high (*n* = 39) GGPSS expression groups. The distribution of the SRC volume is represented by the central line as the median, the shaded area as the interquartile range, and the whiskers indicating the full range of data.

**Supporting Information 2** **Table S1:** Odds ratio for the association between GGPPS expression and simple renal cyst size.

## Data Availability

The data underlying this article will be shared on reasonable request to the corresponding author.
