# Supplementary material for: GGPS1 Promoter Variant (rs3806394) Is Associated With Larger Simple Renal Cysts via Reduced GGPPS Expression
Source: Hum Mutat. 2026 Mar 5;2026:8347509. doi: 10.1155/humu/8347509 (PMC12961355; doi:10.1155/humu/8347509)
Supplement: Supplementary file 1 — Supporting Information 1 Figure S1: (a) Representative IHC staining images of GGPPS in SRC tissues. (b) Violin plot showing the SRC volume (cm3) for the low (n = 38) and high (n = 39) GGPSS expression groups. The distribution of the SRC volume is represented by the central line as the median, the shaded area as the interquartile range, and the whiskers indicating the full range of data. [file HUMU-2026-8347509-s001.pdf]

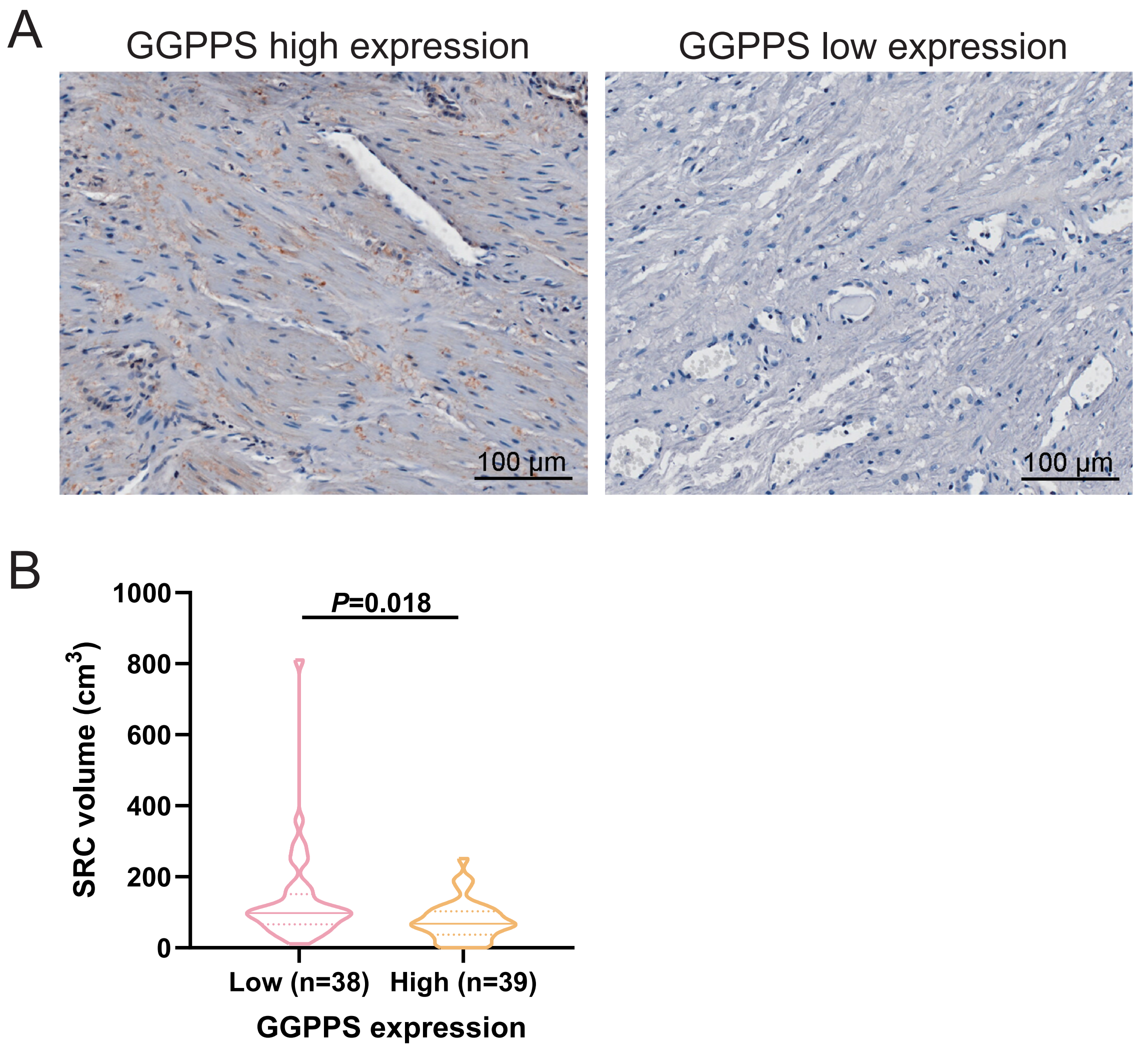

Figure S1. (A) Representative IHC staining images of GGPPS in SRC tissues. (B) Violin plot showing the SRC volume (cm³) for the low (n = 38) and high (n = 39) GGPSS expression groups. The distribution of the SRC volume is represented by the central line as the median, the shaded area as the interquartile range, and the whiskers indicating the full range of data.
