# Supplementary material for: GGPS1 Promoter Variant (rs3806394) Is Associated With Larger Simple Renal Cysts via Reduced GGPPS Expression
Source: Hum Mutat. 2026 Mar 5;2026:8347509. doi: 10.1155/humu/8347509 (PMC12961355; doi:10.1155/humu/8347509)
Supplement: Supplementary file 2 — Supporting Information 2 Table S1: Odds ratio for the association between GGPPS expression and simple renal cyst size. [file HUMU-2026-8347509-s002.docx]

**Table S1. Odds ratio for the association between GGPPS expression and simple renal cyst size.**

| Variable | Crude Model | | Model 1 | | Model 2 | |
| --- | --- | --- | --- | --- | --- | --- |
|  | OR (95% CI) | *P* value | OR (95% CI) | *P* value | OR (95% CI) | *P* value |
| Age (years) | 1.05(1.00-1.09) | 0.057 | 1.04(0.99-1.09) | 0.124 | 1.06(1.00-1.13) | 0.053 |
| Gender (Female vs Male) | 0.50(0.20-1.25) | 0.504 | 0.51(0.19-1.35) | 0.174 | 0.50(0.18-1.40) | 0.186 |
| BMI (kg/m²) | 0.93(0.81-1.08) | 0.328 | 0.91(0.77-1.07) | 0.263 | 0.92(0.76-1.10) | 0.350 |
| Systolic BP (mmHg) | 1.00(0.97-1.02) | 0.862 | - | - | - | - |
| Diastolic BP (mmHg) | 1.01(0.96-1.05) | 0.809 | - | - | - | - |
| Number of cysts (1 vs ≥2) | 1.17(0.48-2.88) | 0.726 | - | - | 0.50(0.10-2.58) | 0.408 |
| Location (Bilateral vs Unilateral) | 1.16(0.47-2.87) | 0.742 | - | - | 1.41(0.32-6.33) | 0.652 |
| Hypertension (Yes vs No) | 1.16(0.47-2.87) | 0.742 | - | - | 0.95(0.28-3.25) | 0.933 |
| Diabetes (Yes vs No) | 1.03(0.30-3.53) | 0.961 | - | - | 0.82(0.19-3.60) | 0.791 |
| CKD (Yes vs No) | 0.32(0.03-3.26) | 0.339 | - | - | 0.16(0.01-2.07) | 0.162 |
| Kidney stones (Yes vs No) | 1.03(0.27-3.89) | 0.965 | - | - | 1.16(0.25-5.35) | 0.853 |
| GGPPS expression (Low vs High) | 3.06(1.21-7.75) | 0.018 | 3.35(1.22-9.15) | 0.019 | 3.79(1.28-11.18) | 0.016 |

Crude Model was unadjusted any covariates;

Model 1 was adjusted covariates including age, gender, and BMI;

Model 2 was adjusted covariates including age, gender, BMI, number of cysts, location of cysts, hypertension, diabetes, CKD and kidney stones);

Abbreviation: OR, odds ratio; CI, confident interval; BMI, Body mass index; BP, Blood pressure; CKD, Chronic kidney disease.
